# Soil Deformation after Water Drop Impact—A Review of the Measurement Methods

**DOI:** 10.3390/s23010121

**Published:** 2022-12-23

**Authors:** Rafał Mazur, Magdalena Ryżak, Agata Sochan, Michał Beczek, Cezary Polakowski, Andrzej Bieganowski

**Affiliations:** Institute of Agrophysics, Polish Academy of Sciences, Doświadczalna 4, 20-290 Lublin, Poland

**Keywords:** soil erosion, splash erosion, crater, soil surface deformation, drop impact

## Abstract

Water erosion is an unfavorable phenomenon causing soil degradation. One of the factors causing water erosion is heavy or prolonged rainfall, the first effect of which is the deformation of the soil surface and the formation of microcraters. This paper presents an overview of research methods allowing the study of microcraters as well as the process of their formation. A tabular summary of work on the measurements of various quantities describing the craters is presented. The said quantities are divided into three groups: (i) static quantities, (ii) dynamic quantities, and (iii) dimensionless parameters. The most important measurement methods used to study crater properties, such as (i) basic manual measurement methods, (ii) photography, (iii) high-speed imaging, (iv) profilometers, (v) 3D surface modelling, and (vi) computed tomography (CT) and its possibilities and limitations are discussed. The main challenges and prospects of research on soil surface deformation are also presented.

## 1. Introduction

Soil is an important part of the biosphere, enabling plant growth and providing habitats for numerous animals and bacteria [1,2,3]. Owing to its retention properties, soil plays an important role in the natural water cycle [4,5,6]. Furthermore, the processes occurring in soil are crucial for the biogeochemical cycle of many other chemical substances and elements [7,8,9,10] and for climate regulation [11]. It is a complex three-phase system (water-air-solids) of great importance to the environment, while at the same time it is highly exposed to various types of deterioration, both mechanical and chemical. One of the processes that can cause physical soil degradation is erosion. Various types of erosion can be distinguished, including water erosion and wind erosion [12]. Water, which on the one hand is essential for human life and the functioning of the environment, can also cause a lot of damage [13,14]. Water erosion can cause changes to the soil surface on several different scales: from splashes during the impact of raindrops to surface runoff during snowmelt or intense rainfall. The rate of the erosion phenomenon is determined by many soil properties, such as texture, aggregate size and stability, and soil moisture [15,16,17].

Water erosion takes many forms, ranging from the impact of a single raindrop through sheet and rill erosion to the formation of deep gullies. The nature of the erosion phenomena affects the way they are studied. Erosion research has been carried out on a large scale, including the study of mass transport and processes in entire catchments [18,19,20,21,22]. On the other hand, with the development of measurement techniques, the scope of possible micro-scale laboratory tests has also increased. This type of research is usually carried out using rainfall simulators or single drops, and it focuses on, among other things, the splash phenomenon, i.e., the first stage of water erosion [23,24,25,26,27,28].

Terry [29] distinguished five main splash subprocesses: aggregate breakdown, cratering, splashing, splash saltation, and splash creep. One of the splash effects is the deformation of the soil’s surface. The craters created by the drops are the result of the ejection, shifting and compaction of the soil at the impact area. Crater formation absorbs a significant amount of falling droplet energy, accounting for an important part in the energy balance of the splash phenomenon [30,31]. In a broader context, the study of craters may be important in determining soil erodibility, modelling the course of water erosion processes, analyzing the effect of precipitation on surface sealing and changes in soil infiltration parameters, the relationship of craters to the ejection of soil particles, and thus the transport of bacteria and pathogens. However, the small scale of the deformation (the dimensions of the craters are defined in millimeters) and the high dynamics of the phenomenon, complicate the investigation. Overcoming the above difficulties is possible by implementing modern measurement methods and being aware of their advantages and disadvantages.

The aim of this work was to provide an overview of the research methods allowing the study of the microcraters as well as the process of their formation. The advantages and limitations of the described methods are discussed, the prospects for further research and the possible directions for the development of measurement systems are outlined.

## 2. Review of Work Focusing on Soil Deformation by the Splash Phenomenon

**Figure 1:**
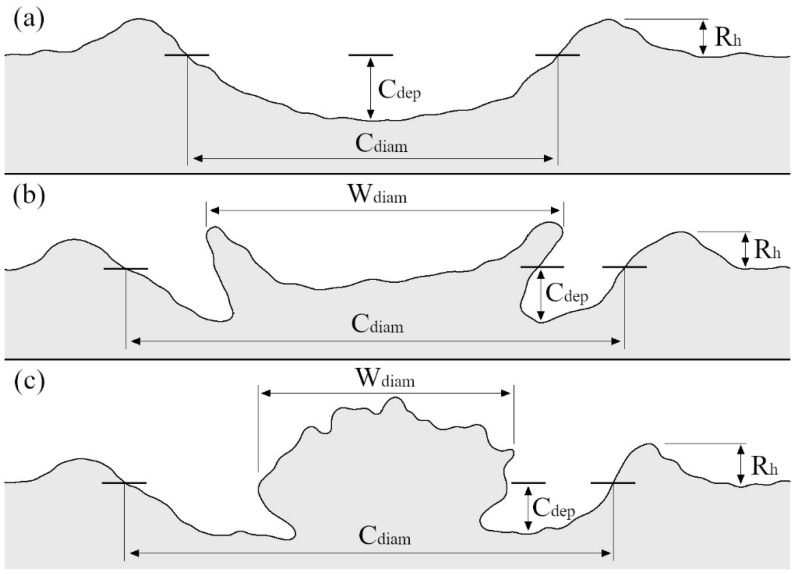
Illustrative diagrams showing examples of crater cross sections ((**a**)–simple crater; (**b**)–crater with spread liquid-grain residues; (**c**)–crater with aggregate liquid-grain residues), with example quantities used to characterise their morphology (C_dep_–depth of the crater; C_diam_–diameter of the crater; R_h_–height of the rim; W_diam_–diameter of the moistened volume of the deposit material).

Information from Katsuragi [31], Marzen and Iserloh [32], and Zhao et al. [33], showed how varied the shapes of craters can be after drop impact (Figure 1).

Craters, characterized by the simplest structure, are formed by a small cavity (with a shape simplistically resembling a hemisphere), which may be surrounded by displaced material forming a rim (Figure 1a). Such deformations are most often observed in moist deposits, which relates their morphology to the interaction of the drops with the water in the pores of the sample. The regularity and depth of the cavity, as well as the height of the rim, depend on the parameters of the deposit, e.g., moisture content, compaction, slope, particle size distribution and the energy of the drops.

Craters formed on dry materials take on more complex shapes (Figure 1b,c). The outer element of the deformation is the rim, but the central part of the cavity is occupied by a form composed of bed grains that were wetted and “glued” by water from the drop. Various nomenclatures were used to name this wetted sample material: nucleus structure granule nucleus [34], liquid-grain residues [35], and granule [36]. Another term that functions in the literature is liquid marble; this phrase refers to a residue of the drop surrounded by hydrophobic deposit grains that impede the mixing of the liquid with the sample material [37,38]. Craters and the moistened material can have different sizes, circularity, and shapes [33,39], which depend on the aforementioned drop energy and deposit parameters.

Difficulties related to the monitoring of crater development arise for several reasons: (i) the sizes of the craters created by the impact of a water droplet is very small (in the order of millimeters); (ii) the process of crater formation is very fast (the time for the deformation to reach its final shape is measured in fractions of a second); and (iii) the displacement process involves two-phase materials (soil particles from the sample and droplets from the broken drop and/or from wet soil).

The abovementioned difficulties can be solved (at least to some extent) by using measuring instruments/systems that are appropriate to the specificity of the measurement. These instruments/systems allow the determination of quantities which can be divided into three groups: (i) static quantities–relating to the dimensions of the crater (such as the depth and diameter of the craters expressed in units of length); (ii) dynamic quantities–making it possible to characterize the process of deformation (such as time taken to achieve the final crater geometry and if its observation is possible, or the stages of reaching this final form expressed in units of time); and (iii) dimensionless parameters–which most often are relative values, i.e., they are the ratio of two or more quantities (for example the ratio of crater depth to its diameter), and then the collected information can be presented more universally, allowing a comparison of results from even strongly differing experiments. Taking into account the topic of this article, which focuses on measurement methods, the issues discussed further in the article relate to the first two groups of quantities.

Regarding the first group, such as the depth and diameter of microcraters (Table 1), it should be emphasized that the results often depend on the methodology adopted for the measurement. For example, crater diameter can be measured based on the rim-to-rim diameter or at the level of the surface before the drop impact–see Figure 1. For both measurements different results will be obtained, therefore, in publications describing the size of the craters, it is necessary to clearly define what the authors mean by a given term. Other static quantities often used to characterize emerging craters are the inclination of the crater walls, the dimensions of the rims around the impact point, the dimensions of the wetted volume of the deposited material, and the crater surface area or crater volume. For a better understanding of the discussed quantities, some of them were shown in Figure 1.

Concerning the group describing the dynamics of crater formation, it includes, among others, the time of achieving the final crater geometry, the time of reaching the crater’s maximum diameter, and the dynamic of change in dimensions, such as the depth or slope of the walls at different stages of the deformation process.

Table 1 provides a summary of the works, which take into account the measurement of craters formed after the drop impact. The broader problems of the listed works were mostly related to issues of soil erosion or the mechanics of powdery materials. In the case of microcraters, it was the main research subject or appeared as a complement to the main theme. Some works used soil [40,41], others used materials that could serve as a soil model, i.e., sand [42] or glass beads [39,43]. Differences between experiments also relate to the size of the drops or the height of their fall, resulting in energy changes at the moment of impact [30,44]. The above issues affect the deformation shape and the spectrum of determined quantities.

A factor that could affect the scope of the determined quantities was often the limitations of the measurement equipment, reducing some of the research to qualitative observations. The carrying out of quantitative observations has been facilitated by the spread of advanced measurement techniques (such as high-speed cameras), which have had a significant impact on the increase in the number of publications on crater formation after drop impact (the vast majority of the papers included in Table 1 are from the last 15 years).

## 3. Crater Measurement Methods

**Figure 2:**
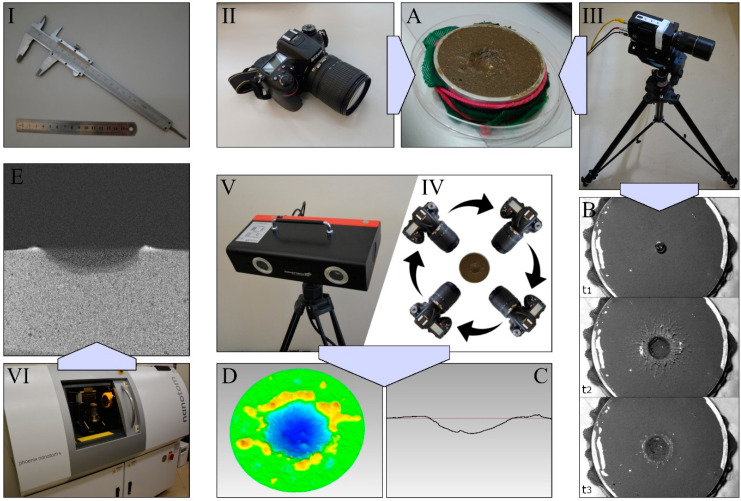
Measurement equipment and methods of analysing data acquired with its help. Measurement equipment: (**I**)–basic manual measurement methods using, among others, a ruler or calliper; (**II**)–camera; (**III**)–high-speed camera; (**IV**)–devices making it possible to record images for photogrammetry (e.g., cameras); (**V**)–surface scanners and profilometers; (**VI**)–computed tomograph. Data analysis methods: (**A**)–photo analysis; (**B**)–record analysis; (**C**)–profile analysis; (**D**)–3D surface model analysis; (**E**)–tomographic image analysis.

Advances in technology have allowed for the use of new devices with increasing accuracy, and extended the measurement possibilities in crater research. The currently used methods were divided into groups and described below. The criterion for division was the type of raw data acquired in the measurement and the scope of information that can be obtained after their analysis (Figure 2).

### 3.1. Basic Measurement Methods

Many early studies on craters formed after a drop impact were based on qualitative analysis. Terry [29] reported that on rip-ploughed soil the craters were narrow and deep, while on the other hand, with burned soil, the craters were shallow and wide. Al-Durrah and Bradford [40], found that cavities formed on more compacted samples had a smaller volume than those formed on loose samples, which was due to smaller depths of deformation. In Ryżak et al. [51], information on craters, complementing the main theme of the study, was the confirmation of their occurrence.

The most basic method for a quantitative analysis of craters involves using a ruler or calliper. With their help, the diameter and depth of the deformation can be determined [30]. The small size of the craters formed after a drop impact makes it difficult to consider such measurement as accurate; moreover, the manual nature of the method causes a high risk of damaging the craters.

Another way to measure deformation is to carry out experiments that analyse the phenomenon based on properly prepared beds formed from several layers differing in colour. This approach may provide information about the displacement of the bed elements located at particular areas and make it possible to estimate the depth of bed penetration by the drop. Atherton et al. [61] prepared beds of glass beads with layers varying in wettability and color and analyzed their interaction with the drops, after impact. In the work of Mazur et al. [58], deposits of glass beads of different colours were used to analyze, among other things, their displacement and the origin of the particles forming the area wetted by the drop. The downside of the method is the difficulty in determining the accuracy of deposit preparation (for example, thickness of layers), limitations due to the size of the beads, as well as the amount of work required to prepare a single sample. Additionally, the use of soil in samples of this type can raise significant problems concerning the technical issues of preparing the deposits, but also the substantive justification for their use.

### 3.2. Photography

Photo analysis requires capturing a clear image of the surface deformed by the drop impact using any device that allows it, such as a camera. The measurements in question focused on the final shape of the craters (the high-speed imaging technique used to analyse the dynamics of cratering was considered in the next section). To minimize the inaccuracy of the dimensions presented in the images, the device used should be positioned over the crater, as perpendicular as possible. Analysis of the collected data can be carried out in any graphics program with the option to measure the distance between two points in a photograph.

Ellison [62] and Mazurak, and Mosher [63], used photographs of samples to present the results of experiments on sample surface change after precipitation. The work of Katsuragi [49], Geißler et al. [64], and Tarasenko et al. [65] shows that today photographs are also commonly used in a similar way. The images can be the basis for analyzing and describing the morphology of the deformation, helping to determine the regularity of the craters, the shape of the wetted material and the presence of rims. For example, Ahn et al. [23] and Mazur et al. [58] used photographs to analyze the area and shape of sample material wetted by drops. However, in most cases, analysis of photographs has served to determine the diameter of the craters, usually making it possible to determine the approximate width of the rim; such measurements were made, e.g., in the works of Delon et al. [43] and Matsuda et al. [55].

The advantage of photo-based analysis is its simplicity. Images of craters are recorded quickly, and measurements can be made in both laboratory and field conditions. There are a large number of programs that allow work to be conducted on the collected data, and the processing is also not complicated in most cases. To ensure the quality of the data, it is worth using equipment that guarantees the high resolution and acuity of the recorded images. Furthermore, by eliminating the need for equipment to be in contact with the surface of the samples during measurements, analysis based on photos removes the risk of damaging the craters. The method can be useful in studies involving a large number of repetitions or variants, where only the static quantities of the craters are determined.

The major disadvantage of photographic analysis is the limitation of the information obtained. Noticeable is the inability to determine the depth of the investigated deformations. An additional problem can be the time-consuming aspect of the research when the processing of images in a program is done manually. The difficulties in precisely determining the edges of craters and rims can also complicate analysis.

### 3.3. High-Speed Imaging

A development of the measurement method based on photography is the analysis of videos, which are, in practice, sequences of consecutive images. High-speed cameras are used to record dynamic phenomena, such as the splash phenomenon. The positioning of the camera should make it possible to observe the process of deformation and determine the intended quantities, which usually means placing the equipment as perpendicular as possible to the surface of the sample, over the drop impact point. Parameters determining the quality of recordings include the number of frames captured per second and the associated exposure time (the higher the number of frames and lower the exposure time, the more precisely moving objects are captured), the depth of field (which determines the size of the area in which the image is sharp) and the resolution of the records.

The popularity of high-speed cameras is partly due to the development of recording and processing techniques, which are now fully computerized. The recordings can be processed in programs dedicated to a specific type of device, or they can be extracted as frames and analysed by commonly available graphics programs. Before the availability of modern technical solutions, an analysis of the collected data was more difficult–in Ghadiri [30], the use of high-speed cameras to describe the impact of a drop on a water target involved projecting a sequence of successive images onto graph paper and outlining the contours of the analyzed shapes (described as corona and crater).

Based on recordings from high-speed cameras, it is possible to obtain data on the dynamics and course of the cratering phenomenon [40,44,53,56]. Some works used single images or short sequences of images taken by the method of high-speed photography as an independent object of analysis or a way of presenting successive stages of the studied process [42,43,45,49,54]. The quantities determined based on the films include, among others, the time of crater formation and the change in its diameter over time, which was considered in the work of Delon et al. [43], Nefzaoui and Skurtys [44], Zhao et al. [53], and Mazur et al. [58]. The course of the wetting of deposit material by drops can also be the subject of research [36,38,50,54]. Camera measurements provide insight into the movement of bed material [42,48] and the formation of the rim [39,44,49].

In addition, high-speed camera recordings make it possible to precisely define the place of drop impact, which can affect the results of experiments that take into account the use of samples with irregular, rough surfaces. The use of additional cameras whose optical axes are at the level of the sample makes it possible to extend the analysis of craters by monitoring the parameters of the falling drop (information regarding the trajectory of particles ejected by splash or the process of corona formation may also be an added value). Referring to Range and Feuillebois [66], for low heights, i.e., several dozen centimetres, the law of conservation of energy can be used to estimate the drop velocity, but for greater heights the drag caused by air becomes so significant that it is worthwhile to use high-speed cameras or other devices to monitor the velocity of the drop immediately before it impacts the sample.

Measurements taken with high-speed cameras also have limitations. The main disadvantage is the inability to determine the depth of the craters. Appropriate equipment for the stand can also be a problem, mainly the total cost of cameras, lighting and software. Note the difficulty of using cameras in the field; these devices are so far mostly laboratory equipment.

### 3.4. Profilometry

As stated above, the main limitation of the methods based on photos and recordings is the inability to determine crater depth and rim height. On the other hand, the use of a ruler or calliper is associated with low precision and the risk of damaging the deformations, resulting in errors affecting the obtained results. These complications have led to a search for a way to measure crater depth with non-contact methods and high accuracy. An example of a device that makes it possible to obtain the desired results is a laser profilometer, with which one can obtain the deformation profile, i.e., a two-dimensional depiction of the interior of the crater. During the measurement, the laser beam emitted by the device is pointed at the target, and the way it is reflected provides information about the structure of the investigated surface. The results are obtained based on the interference of the measurement beam with a reference beam of known optical path or the reflection angle of the measurement beam (trigonometric triangulation). The advantages of laser profilometers are high accuracy, resolution and the speed of measurement. Profilometers can be used in both laboratory and field research. An analysis of the results is usually performed in programs dedicated to specific devices.

Another method of obtaining profiles was presented in the works of Zhao et al. [33] and De Jong et al. [35]. Information about the shape of the deformation was based on an analysis of recordings made with high-speed cameras on which the distortion of laser beams projected onto the sample during the impact of the drop was recorded. To obtain the profiles, one can use the measurement methods standardly applied to prepare a 3D model of the deformation, which are discussed later. The issue determining the use of the full capabilities of these techniques or being limited to a single profile interpretation is the scope of information relevant to the user.

Profile analyses were used to determine the depths and diameters of the craters in the works of Ghadiri [30], Long et al. [42], Beczek et al. [41], and Mazur et al. [58]; they also found application in determining the heights of the rims [41] and the slope of the crater walls [35]. In the aforementioned works by Zhao et al. [33] and De Jong et al. [35], through the additional use of high-speed cameras, the dynamics of the craters’ shape changes after the drop impact were determined, allowing insight into the process of their formation. De Jong et al. [35] also investigated a process called avalanche (the displacement of deposit grains from the rim and walls of the crater to its center), the occurrence of which can affect the final diameter and depth of craters.

The main disadvantage with measurements based on crater profiles lies in conducting analyses of three-dimensional objects (which craters are) based on their two-dimensional, often single depiction. The use of a single profile does not provide a chance to accurately determine the volume of craters, thereby comparing it with, for example, the volume of a drop. Admittedly, there are known attempts to calculate the volume of craters based on their depth and diameter obtained from profiles using the hemisphere formula [30,41]. However, using the above equation results in an overestimation of the crater’s volume [60]. In addition, without a complete picture of deformation, it is difficult to be certain of carrying out the profile through key points, i.e., those where the crater is deepest, the diameter representative, and where the rim reaches its greatest height. Another problem related to the limitations of the method (at least for some of the devices) relates to the difficulty of making a profile of craters of a more complicated shape, in the center of which is an irregular material wetted by the drop, obstructing access of the laser beam to part of the deformation (Figure 1b,c).

### 3.5. 3D Surface Modelling

Measurement methods that involve making digital 3D models of craters are most often based on structured light scanning, laser scanning, or photogrammetry. The 3D models can also be built from profiles arranged side-by-side made with profilometers [30,39,49].

In the case of photogrammetry (called structure from motion), the preparation of a 3D model assumes using a set of images of the examined object. Photographs should be taken from different angles and merged in software dedicated for this purpose. Photogrammetry measurements do not require any specialized equipment. The images can be taken with a camera, as in the work of Long et al. [42] and Laburda et al. [67], or be frames cut from a video, as in Vinci et al. [68,69]. For measurements outside the laboratory, one should consider using equipment that provides shade from sunlight and uniform artificial lighting. Photographs that form the basis of the method must be of good quality and high resolution (the self-timer function, for example, can be helpful in order to exclude the shaking of the device that occurs during manual shooting, thus ensuring better quality of the recorded data). The processing of images can be facilitated by placing markers on or near the object to help identify characteristic points, a method that is related to the crucial phase of the process referred to as image rectification, which consists of projecting the pictures onto a common plane.

3D scanners are devices designed to create digital models of objects. The resolution of measurements made with 3D scanners can be as high as a few dozen micrometres, and in addition, the measurement time is usually tens of seconds. This type of equipment is based on laser scanning technology or structured light scanning. In the case of laser scanners, the object is illuminated by a red or blue laser beam(s). Structured light scanners (using white, blue or green light) project a pattern onto the examined object, usually consisting of a series of stripes of varying width which change their position during the measurement. Both types of devices measure based on the way the emitted light is reflected from the tested object, usually by the angle of reflection registered by the detector [70,71,72,73]. Scanners can be in stationary form (set on a tripod, working together with a rotary table that changes the position of the sample during the measurement) or hand-held.

The possibilities offered by analysing 3D models are similar, regardless of the method used to obtain them. The preliminary result of the measurement is usually a point cloud (or several point clouds, when capturing the details of the deformation requires repeating the measurement from different sides) representing the geometry of the studied object. The point cloud requires further processing depending on the equipment and software used. Processing may include removing measurement errors, the calibration of several point clouds with each other and their final merging and transformation into a triangular mesh model on which proper crater dimension analyses can be conducted. 3D model analyses may involve the application of reference surfaces that provide a comparison point for deformation [72]. A reference surface can be a 3D scan of the sample surface before the drop impact or an artificially imposed surface on the model to represent the level of the sample before the deformation.

The accuracy of the resulting model depends on the technique and equipment used. Aguilar et al. [74] compared the applicability of photogrammetry and surface scanners in the context of soil relief measurements under field conditions. It was found that both methods allowed the fast generation of digital models providing good accuracy, which was, however, better with the laser scanner, which enabled the imaging of small aggregates and the spaces between them. 3D models provide the opportunity to determine the highest and lowest points within the entire deformation (not just in a single two-dimensional profile)–as was done in the work of Mazur et al. [60]–or to present the surface roughness within the entire crater [35,39,42,49,52]. In addition, it is possible to measure the diameter, the circularity and volume of craters, the quantities that characterize the rim, including the volume of material that forms it, and determine the dimensions of the material wetted by the drop if such element is present and measurable [56,60]. In the case of Laburda et al. [67], the use of photogrammetry made it possible to determine the change in level and roughness of soil samples after natural precipitation. 3D scanners were also used to describe the surface after simulated rainfall [75] and assess soil loss due to erosion [76].

It should be noted that, regardless of the technology, scanners have a problem when it comes to measuring transparent surfaces (a similar problem can occur with moist soil samples) or objects exposed to strong illumination, such as sunlight [70,73]. Although, a change in the wavelength emitted by modern lasers makes illumination a decreasing problem. For some scanners, measurement complications can occur with objects whose colour is close to black [77]. A potential problem may also be the limited measurement volume of the equipment, which in the case of larger samples may require combining several scans. As with profilometers, measurements can be hampered by the more complex shape of the crater (Figure 1b,c), where it can be difficult to acquire data on parts of the deformation area.

### 3.6. Computed Tomography (CT)

Computed tomography scanning is a measurement technique that involves generating images of the interior of objects with the use of X-ray radiation. Depending on the physical properties of the sample material (especially its density), the radiation emitted by the X-ray source in the device is absorbed to a greater or lesser degree, and the part that passes through the sample is recorded with detectors placed behind the studied object [78,79,80]. The sample image is obtained by using mathematical algorithms incorporated into the device’s software.

Computed tomography allows the determination of static qualities, characterizing craters with the highest accuracy among the methods mentioned herein. The advantage of tomography is that one can obtain cross-sections of samples, making it possible to analyze the arrangement of material inside the sample (not, as in the case of profiles, only on the surface). Beczek et al. [41] described the use of X-ray microtomography to determine the diameter, the depth of crater and the height of the rim formed on soil samples after the impact of a single drop. The same methodology was applied by Mazur et al. [58] in a study of the splash phenomenon on beds of glass beads. In both cases, measurements were based on cross sections of prepared samples. Yang et al. [81] used synchrotron-based X-ray microcomputed tomography to study porosity changes in a soil sample caused by drop impact.

The design of the measuring equipment is such that computed tomography research can only be performed in the laboratory, and the size of the analyzed samples is limited [82]. The cost of purchasing and exploiting the apparatus can be another obstacle to using the technique. A disadvantage of using the method is the need to transport the samples to the tomograph, which can affect the bed arrangement after the drop impact. A rather long scanning time, in the order of several minutes [41], can also be a complication, affecting the changes in the arrangement of the sample caused by evaporation of water.

## 4. Potential Use of Crater Measurement Methods in Water Erosion Research

Water erosion occurs in many forms such as splash, sheet, rill and interrill erosion, gully erosion, bank erosion, or snowmelt erosion [83], differing in the mechanism of formation and the scale of the phenomenon. Due to the widely varying size scales, measuring each of the above-mentioned phenomena requires the selection of appropriate measurement tools. Some of the methods described earlier as methods for the measurement of craters find their application in the wider range of water erosion studies.

As Kinnell (2005) [83] points out, erosion is a process that involves the detachment of soil material from the surface of the soil matrix followed by the subsequent transport of the detached material away from the initial location. To quantify this transported soil material, high-speed cameras are successfully used to determine the angle of ejection; ejection velocity; range (displacement); and maximum height to which the soil particles have risen [84] These experiments, due to the requirements for adequate lighting and system calibration, are mainly carried out under laboratory conditions using single drops of simulated precipitation.

While high-speed cameras are successfully used in splash erosion studies and can also be used in the analysis of surface runoff, they have limitations in field experiments resulting from their small field of observation and the need to mount them and ensure appropriate lighting. These limitations do not apply when analyses of photo sequences taken with hand-held or drone-mounted cameras and/or ultralight aircraft are used. Both the height from which the photos are taken and their frequency will depend on what exactly is to be observed. In any case, modern software for image analysis allows for obtaining a large amount of data useful for interpreting the phenomenon of water erosion.

The microtomograph is another method used to measure soil erosion processes. Due to the “layered” imaging and cross-sectioning of the sample, it is possible to analyse the compaction of the sample that occurs as a result of droplet impact. This method can be used to estimate the energy that has been expended in compacting the soil at the point of impact of the drop. The application of this technique has certain limitations, mainly related to the size of the sample itself as well as the need to place it in the apparatus, which makes it impossible to carry out measurements under field conditions.

Another measurement method used for microcraters that can also be used for erosion measurements is the 3D surface scanner. The scanners used today are suitable for measuring both small and large objects. They come in the form of portable devices that can successfully scan the change in soil surface after rainfall under field conditions. With their use, it is possible to describe the course of splash, sheet, rill and interrill erosion, or even gully erosion. Similar applications can also be found with profilometers, which are suitable for characterising surface changes on a larger scale.

The applications of the crater measurement methods reported here represent only part of the methods developed over the years to measure particular forms of erosion. The information about the possibilities of using the described methods in the context of erosion aspects beyond craters caused by drop impacts was intended to broaden awareness of their versatility.

## 5. Challenges and Prospects

There are many differences between the measurement methods described in this paper. They refer to the scope and resolution of the acquired data, the character of the measurements, including the time required or method of execution, and finally the cost of the equipment. Considering that equipment choice is one of the first decisions made in planning research, it is important that such decisions be made consciously based on the study objectives, the range of quantities to determine and taking into account all the advantages and disadvantages of the available methods.

One aspect of the development of measurement methods is to solve problems arising from their limitations. In the case of methods for crater measurement, such limitations apply mostly to surface scanners and more specifically to problems caused by inadequate lighting or the type of study surfaces (e.g., measuring craters on moist, dark-colored soils). Considering the growing possibilities of the new devices, it can be concluded that these problems have been recognized and are gradually being solved. For profilometers, the development prospects can be seen in the increasing speed of measurements. Sufficiently advanced devices could provide capabilities analogous to the set-ups used by Zhao et al. [33] or de Jong et al. [35], based on the simultaneous use of a high-speed camera and a laser beam. This makes it possible to simplify similar measurements (even by automating the processing results), and thus could provide easier insight into the change in the linear dimensions of the crater in the process of its formation.

Implementing promising new measurement techniques such as 3D microscopy or high-speed 3D shape measurement, can also be a way to facilitate measurements or expand their scope [73,85,86]. Regarding the first one, 3D microscopes take a sequence of measurements creating–using the device’s software–a 3D image of the sample resembling the result of the use of a scanner. High accuracy and a significant level of measurement automatization appear to be important advantages. The limited sample size associated with the design of the microscopes and the need to use the technology only in the laboratory can be a problem. Regarding high-speed 3D shape measurement, it is a technique that requires further development, but in the long term it may enable the creation of 3D models showing the changes in the morphology of craters occurring in the process of their formation.

A different, important issue, is the adaptation of equipment for field studies. The appropriate construction of equipment could facilitate measurements outside the laboratory, contributing to an increase in the number of works, including field studies.

Current research questions about craters are, among others, related to the beginning of their formation process, and include questions about soil compaction at the point of the drop impact, an analysis of the origin of the ejected material, and the initial position of the material forming the rim. Although these topics were addressed in the works of Mazur et al. [58], de Jong et al. [35], and Katsuragi [39], these studies were conducted on model deposits, mainly glass beads, which reflected in the deficit of works dealing with the above issues in the context of soil.

The potential application of the results from crater analyses includes implementing them into erosion models to better predict the magnitude of the phenomenon, especially in the early stages of precipitation. Data about soil susceptibility to deformation due to drop impact may additionally be an appropriate supplement to erodibility information. The methods presented herein may find application in monitoring splash erosion after natural precipitation, facilitating the calculation of crater volumes, or serving as a proxy for the amount of splashed material. Following Laburda et al. [67], surface deformation analysis using photogrammetry can provide an alternative to conventional measurement methods using standard splash cup. On the other hand, the results of the field measurements of craters could help determine how accurately the trends observed under controlled laboratory conditions translate into results obtained on undisturbed soil after natural precipitation.

## Figures and Tables

**Table 1:** Overview of the work involving measurement and analysis of craters after drop impact ^1^.

Authors	Measuring Method	Measuring Scope						Complementary Description of Measuring Scope
		Qualitative Description of Craters	Diameter of Crater	Depth of Crater	Volume of Crater	Dynamics of Crater Formation	Additional Quantities	
Mihara et al., 1950 [45]	Photography, high-speed photography		X	X	X	X		
Al-Durrah and Bradford, 1982 [40]	High-speed recording	X						Qualitative description of the craters (diameter and depth) and analysis of the mechanism of crater formation
Moss and Green, 1987 [46]	Qualitative observations	X						Qualitative description of craters as complementary information to the main measurements
Terry, 1990 [47]	Qualitative observations	X						Qualitative description of craters as complementary information to the main measurements
Terry, 1998 [29]	Qualitative observations, photography	X						Qualitative description of crater morphology, analysis of the mechanism of crater formation, images of cratering
Ghadiri, 2004 [30]	Surface profiler, basic research methods		X	X	X		X	Additional calculation of crater area, visualisation of the craters’ diversity of shape
Furbish et al., 2007 [48]	High-speed recording						X	Information about the occurrence of craters during the experiments; description of the mechanism related to the formation of craters
Katsuragi, 2010 [49]	High-speed recording, profilometry, 3D surface modelling		X	X		X	X	Mechanism of crater formation, presentation of the shape of craters on profiles and 3D height maps; supplementary dimensionless parameters describing the crater
Marston et al., 2010 [34]	Photography, high-speed recording		X				X	Mechanics of crater formation, analysis of the material moistened by the drop
Delon et al., 2011 [43]	Photography, high-speed recording		X			X	X	Measurement of the diameter of the material wetted by the drop; changes of the crater size during drop impact
Emady et al., 2011 [50]	Photography, high-speed recording					X	X	Analysis of the mechanism of crater formation and the shape of the material moistened by the drop
Katsuragi, 2011 [39]	High-speed recording, profilometry, 3D surface modelling		X	X		X	X	Information on the morphology of the craters; presentation of the shape (depth, diameters) of craters on profiles and 3D height maps; supplementary dimensionless parameters describing the crater
Nefzaoui and Skurtys, 2012 [44]	Photography, high-speed recording		X			X	X	Mechanics of crater formation and description of the material wetted by the drops (including dimensionless coefficients)
Ahn et al., 2013 [23]	Photography, high-speed recording						X	Area, circularity and roundness of wetted perimeters generated by a drop impact
Emady et al., 2013 [36]	Photography, high-speed recording						X	Analysis of the mechanism of crater formation and the shape of the material wetted by the drop
Long et al., 2014 [42]	High-speed recording, profilometry (photogrammetry), 3D surface modelling		X	X		X	X	Mechanics and crater formation, information on rim height and dimensionless parameters for crater description
Ryżak et al., 2015 [51]	High-speed recording	X						Notes on crater occurrence as supplementary information to the main experiments
Zhang et al., 2015 [52]	High-speed recording, profilometry, 3D surface modelling		X			X	X	Mechanics of crater formation, dimensionless parameters describing the crater
Zhao et al., 2015 [53]	High-speed recording, profilometry		X	X		X	X	Mechanics of crater formation, size of granular residues, dimensionless parameter (aspect ratio α equal to depth to diameter ratio)
Zhao et al., 2015 [33]	Photography, high-speed recording, profilometry			X		X	X	Mechanics of crater formation
Supakar et al., 2016 [37]	High-speed recording						X	Considering the importance of craters in the context of the formation of liquid marbles
De Jong et al., 2017 [35]	Photography, high-speed recording, profilometry, 3D surface modelling		X	X	X	X	X	Dimensionless parameters related to diameter, depth and volume of transient and final crater, slope of crater wall
Beczek et al., 2018 [41]	Profilometry (microtomography)		X	X	X		X	Crater aspect ratio α equal to depth to diameter ratio, height of the rim
Lardier et al., 2019 [54]	High-speed recording, 3D surface modelling		X			X		Mechanics of crater formation, dimensionless parameters
Matsuda et al., 2019 [55]	High-speed recording		X			X		Dynamics of the crater formation process after the impact of hydrogel spheres
Padmanathan et al., 2019 [38]	Photography, high-speed recording						X	Investigating the importance of craters in the context of the formation of liquid marbles
Wyser et al., 2019 [56]	High-speed recording, profilometry, 3D surface modelling		X	X	X	X	X	Mechanics of crater formation, distributions of deposited volume; presentation of diameters, depths of craters and rims heights on profiles; dimensionless parameters
Matsuda et al., 2020 [57]	Photography, high-speed recording					X	X	Analysis of the origin of material displaced within the crater; dynamics of the crater formation process after the impact of hydrogel spheres
Mazur et al., 2020 [58]	High-speed recording, profilometry		X	X		X	X	Analysis of the origin of material displaced within the crater; shape of the material wetted by the drop; rim size, mechanics of crater formation
De Jong et al., 2021 [59]	High-speed recording, profilometry		X	X	X			Dimensionless parameters related to diameter, depth and volume of crater
Mazur et al., 2022 [60]	3D surface modelling		X	X	X		X	Circularity of crater

^1^ For simplicity of overview, all analyses based on profiles regardless of the method of obtaining them (profilometers, photogrammetry, 3d scanners, microtomography) have been called profilometry. The mark “X” indicates the papers in which these quantities were measured.

## Data Availability

Not applicable.
